# Incidence and Clinical Implications of Anatomical Variations in the Pancreas and Its Ductal System: A Systematic Review and Meta-Analysis

**DOI:** 10.3390/life13081710

**Published:** 2023-08-09

**Authors:** Mathias Orellana-Donoso, Daniel Milos-Brandenberg, Andoni Benavente-Urtubia, Javier Guerra-Loyola, Alejandro Bruna-Mejias, Pablo Nova-Baeza, Álvaro Becerra-Farfán, Walter Sepulveda-Loyola, Ricardo Miguel Luque-Bernal, Juan José Valenzuela-Fuenzalida

**Affiliations:** 1 Escuela de Medicina, Universidad Finis Terrae, Santiago 7500000, Chile; mathias.orellana@unab.cl; 2 Departamento de Morfología, Facultad de Medicina, Universidad Andrés Bello, Santiago 8370146, Chile; ambu.1910@gmail.com (A.B.-U.); javier.guerra@unab.cl (J.G.-L.); abrunam@unab.cl (A.B.-M.); pnova@usach.cl (P.N.-B.); 3 Escuela de Medicina, Facultad Ciencias de la Salud, Universidad del Alba, Santiago 8320000, Chile; danielmilos.b@gmail.com; 4 Departamento de Ciencias Química y Biológicas, Facultad de Ciencias de la Salud, Universidad Bernardo O’Higgins, Santiago 8370993, Chile; alvaro.becerra@ubo.cl; 5 Faculty of Health and Social Sciences, Universidad de las Américas, Santiago 8370040, Chile; walterkine2014@gmail.com; 6 Unidad de Anatomía, Escuela de Medicina y Ciencias de la Salud, Universidad del Rosario, Bogotá 111221, Colombia; ricardo.luque@urosario.edu.co; 7 Department of Morphology and Function, Faculty of Health and Social Sciences, Universidad de Las Américas, Santiago 8370040, Chile

**Keywords:** variations anatomical pancreas, ductal pancreas, anatomical variations, pancreatitis, clinical anatomy

## Abstract

Objective: This systematic review analyzes the anatomical variants in the pancreas and its ductal system to report on their association with pancreatic pathologies. Methods: We conducted a search of the MEDLINE, Scopus, Web of Science, Google Scholar, CINAHL, and LILACS databases from their inception to July 2023. The methodological quality was assessed with the Anatomical Quality Assessment (AQUA) tool. Finally, the pooled prevalence was estimated using a random effects model. Results: 55 studies were found that met the eligibility criteria. The overall prevalence of pancreas divisum (PD) was 18% (95% CI = 15–21%). The prevalence of PD associated with pancreatitis was 30% (95% CI = 1–61%). Conclusions: An anatomical variant of the pancreas such as PD may be the cause of bile duct obstruction, resulting in various clinical complications, such as pancreatitis. Hence, knowing this variant is extremely important for surgeons, especially for those who treat the gastroduodenal region.

## 1. Introduction

The pancreas is an exocrine and endocrine gland that develops from the fusion of two evaginations of the anterior intestine. Its exocrine portion is primarily composed of pancreatic acini, while the endocrine portion is represented by scattered Langerhans islets within the parenchyma. Macroscopically, it is lobulated and pale yellow in color, weighing between 150 and 200 g in adults, with a horizontal length of 12 to 15 cm. Its anteroposterior diameter ranges from 1 to 3 cm, and its height ranges from 4 to 8 cm, gradually tapering towards the tail [1,2]. It is divided into four parts from right to left: the head, neck, body, and tail. The head passes to the left of the duodenum, behind and to the right of the mesenteric vessels. The neck is located just in front of the mesenteric vessels [3]. The body and tail are oblique, both directed posterosuperiorly and to the left. Due to the absence of a capsule, it is surrounded by a layer of cellulose fatty tissue. The common bile duct passes through the gland from top to bottom, joining the pancreatic ducts through the ampulla of Oddi, and then exits into the major duodenal papilla [4]. The excretory pancreatic ducts include the main pancreatic duct (Wirsung’s duct) and, occasionally, the accessory pancreatic duct (Santorini’s duct), which drain into the major and minor duodenal papillae, respectively. Anatomical variations in these ducts may reflect anomalies in the development and fusion of the pancreatic ducts. The pancreas is fixed in position within the abdominal cavity, held from the posterior wall of the abdomen by its connections with the duodenum and excretory ducts. Thus, the pancreas develops within the thickness of the posterior mesogastrium, separated from the posterior wall by Treitz’s fascia.

The pancreas plays a central role in the digestion, absorption, and metabolism of energy substrates. Its exocrine function is modulated by neural and hormonal signals, including gastrointestinal peptide hormones [5]. Due to the lack of basal membranes or compartmental capsules, the islet cells are interspersed within the acini. Therefore, acini located near the islets are called peri-insular acini, while those extracted from the islets are called teleinsular acini. Mourad et al. (1994) mention that some islet cell secretory products, such as insulin, interact with acinar cells and, thus, regulate acinar function [3]. The unique morphology of peri-insular acini is reflected in the presence of high concentrations of insulin in the region [6]. Surgical intervention of the pancreas remains difficult due to its retroperitoneal location, irregular surface, and close relationships with various adjacent structures. Under these circumstances, three-dimensional (3D) reconstruction through digitization is crucial for improving pancreatic surgical techniques. Among the pathologies that can affect the pancreas is pancreatitis, an inflammatory disease that can be acute, recurrent, or chronic. It can also be classified based on clinical, anatomical, and histological criteria. Some known risk factors for acute pancreatitis include gallstones, alcohol abuse, hypertriglyceridemia, endoscopic retrograde cholangiopancreatography (ERCP), pancreas divisum (PD), intraductal papillary mucinous tumor, autoimmune pancreatitis, and genetic risk predisposition. As a result, pancreatitis can become one of the most complex and challenging conditions for physicians and surgeons [7].

Based on the factors stated above, the objective of this study was to identify the prevalence of pancreatic ductal system variants and their association with pancreatitis.

## 2. Methods

### 2.1. Protocol

This systematic review and meta-analysis were performed and reported according to the Preferred Reporting Items for Systematic Reviews and Meta-analyses (PRISMA) statement [8].

### 2.2. Electronic Search

**Figure 1:**
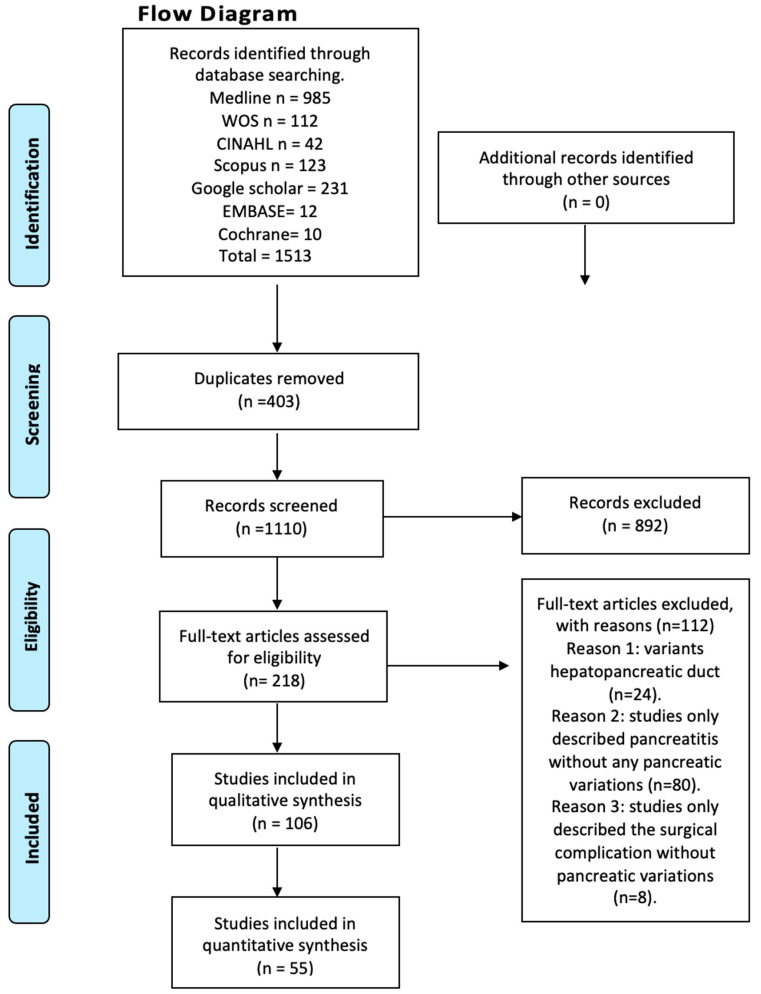
Flow diagram.

We systematically searched MEDLINE (via PubMed), Web of Science, Google Scholar, the Cumulative Index to Nursing and Allied Health Literature (CINAHL), Scopus, EMBASE, Cochrane, and the Latin American and the Caribbean Literature in Health Sciences (LILACS) from inception until June 2023 (Figure 1). The search strategy included a combination of the following terms: “variations anatomical pancreas”, “ductal pancreas”, “anatomical variations”, “pancreatitis”, and “clinical anatomy”, using the Boolean connectors AND, OR, and NOT. The search strategies for each database are available in the supplementary material (Supplementary Table S1). Two authors (JJV and JG) independently screened the titles and abstracts of the references retrieved from the searches. The full text for references that either author considered to be potentially relevant was obtained. A third reviewer (AB) was involved if consensus could not be reached.

### 2.3. Eligibility Criteria

Studies on the presence of variants in the DP and their association with any clinical condition were considered eligible for inclusion if the following criteria were fulfilled: (1) population: samples of dissections or images of the DP; (2) outcomes: prevalence of the DP variants and their correlation with pathologies of the pancreas or its ductal system or surgical complications; additionally, anatomical variants were classified and described based on normal anatomy and classifications proposed in the literature; and (3) studies: this systematic review included research articles, research reports, or original research published in English language databases. Conversely, the exclusion criteria were as follows: (1) population: animal studies; (2) outcomes: prevalence of pancreatic variants; (3) studies that performed variant analyses on other regions of the pancreas; and (4) studies: letters to the editor or comments.

### 2.4. Assessment of the Methodological Quality of the Included Studies

Quality assessment was performed using the methodological quality assurance tool for anatomical studies (AQUA) proposed by the International Evidence-Based Anatomy Working Group (IEBA) [9]. Data extraction and quality assessment were independently performed by two reviewers (JJV and JM). We involved a third reviewer (DM) if a consensus could not be reached. The agreement rate between the reviewers was calculated using kappa statistics.

### 2.5. Data Collection Process

Two authors (AB and E) independently extracted data on the outcomes of each study. The following data were extracted from the original reports: (i) authors and year of publication, (ii) country, (iii) type of study, (iv) sample characteristics (sample size, age, distribution, and sex), (v) prevalence and morphological characteristics of MS, (vi) statistical data reported by each study, and (vii) main results.

### 2.6. Statistical Methods

To analyze the prevalence, we used the Jamovi software (Beta version). The Jamovi project was founded to develop a free and open statistical platform that is intuitive to use and can provide the latest developments in statistical methodology. At the core of the Jamovi philosophy is that scientific software should be “community-driven”, where anyone can develop and publish analyses and make them available to a wide audience; it should be noted that Jamovi is the name of the software and is not an abbreviation [10]. Due to the high heterogeneity in the prevalence data on MS variations, a random effects model was used. The degree of heterogeneity between included studies was assessed using the chi^2^ test and the heterogeneity (I^2^) statistic. For the chi^2^ test, a *p*-value of less than 0.10, as proposed by the Cochrane Collaboration, was considered significant. Values of the I^2^ statistic were interpreted as follows with a 95% confidence interval (CI): 0–40% indicating no important heterogeneity, 30–60% indicating moderate heterogeneity, 50–90% indicating substantial heterogeneity, and 75–100% indicating a significant amount of heterogeneity.

## 3. Results

After conducting a systematized search of the literature, a total of 214 studies were found in the databases reviewed. Following the application of the first exclusion criterion to the search—articles that did not relate to anatomical variations of the pancreas with clinical complications either in the title or in the abstract—a total of 94 studies were left, whose full text were subsequently analyzed. The exclusion criterion corresponding to the type of study was applied (systematic reviews, literature reviews, and letters to the editor), followed by the one related to the content of the articles (articles that speak only of innervation or content variation, and articles in animals), ending with 55 studies included in this review (Figure 1). Furthermore, the included articles were assessed using the AQUA checklist for anatomical studies. The studies included different anatomical variations and clinical considerations.

### 3.1. Description of the Variants Studied

**Figure 2:**
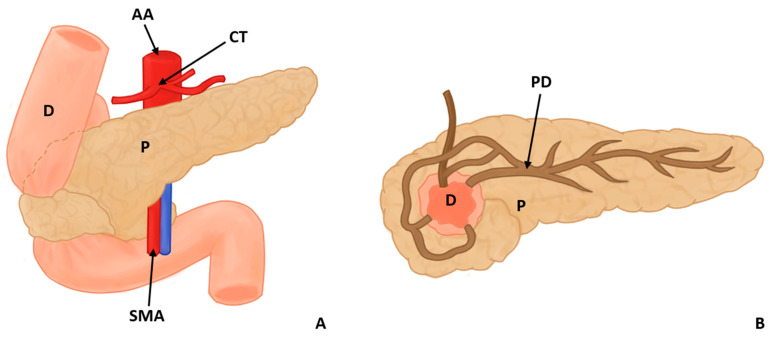
Annular pancreas surrounding the descending portion of the duodenum in 2 views, anterior (**A**) and posterior (**B**) (D: duodenum, P: pancreas, PD: pancreatic duct AA: abdominal aorta, CT: celiac trunk, and SMA: superior mesenteric artery).

**Figure 3:**
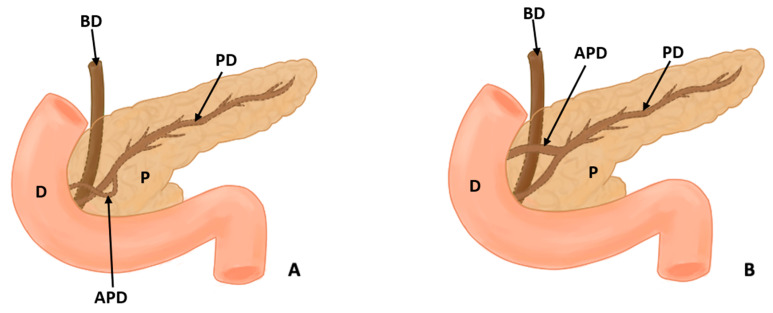
(**A**) shows the course of the accessory pancreatic duct in the pancreatic loop, while (**B**) shows the normal configuration of the pancreatic ducts (D: duodenum, P: pancreas, PD: pancreatic duct, APD: accessory pancreatic duct, and BD: bile duct).

**Figure 4:**
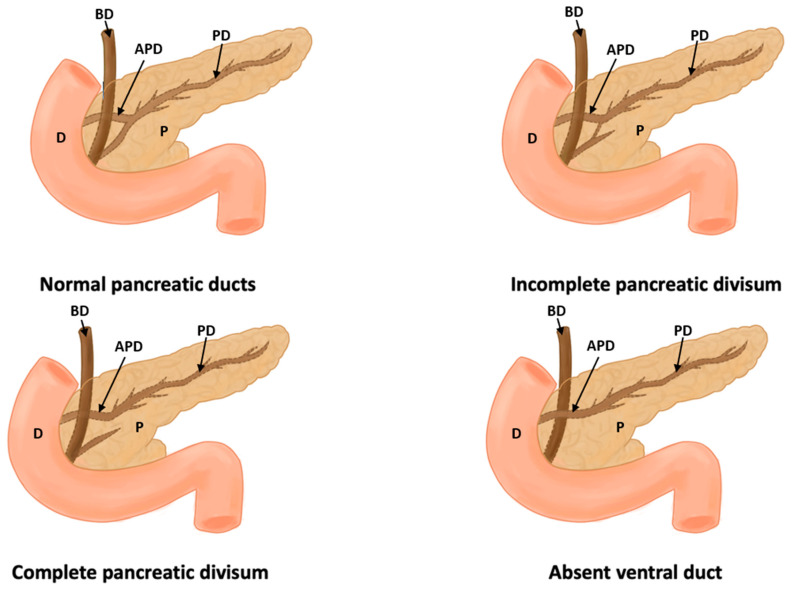
This figure shows a normal pancreas, incomplete pancreas divisum, complete pancreas divisum, and absence of a duct (D: duodenum, P: pancreas, PD: pancreatic duct, APD: accessory pancreatic duct, and BD: bile duct).

In this study, eight types of morphological variants associated with the pancreas were found, which will be described below. Annular pancreas is a rare congenital anomaly characterized by the presence of a pancreatic tissue prolongation that surrounds the second portion of the duodenum [11,12,13,14,15] (Figure 2). Ansa pancreatica is a rare variation of the pancreatic duct where the accessory pancreatic duct makes a sinuous curvature in its course before fusing with the main pancreatic duct due to an obliteration in the accessory duct [16,17,18] (Figure 3). Bifid pancreas is an anatomical variation of the main pancreatic duct where the body and/or tail of the pancreas is duplicated and then fuses or unites in the head of the pancreas, forming a single main pancreatic duct that will continue its normal path towards the greater duodenal papilla [2,19,20]. Circumportal pancreas (CP) is a congenital anomaly of the pancreas where the portal vein is surrounded by normal pancreatic tissue, and, in some cases, the CP may surround the superior mesenteric vein [21,22,23,24,25,26]. PD is one of the most common pancreatic variations, which is produced by an embryological failure in the fusion and rotation of the ventral and dorsal pancreatic buds, which occurs between the sixth and seventh weeks of gestation, causing variations in the pancreas’s ductal system. It can be found in a complete (classical) form, in which the pancreatic secretion is drained through the accessory pancreatic duct, or an incomplete (partial) form, in which there is communication between the ducts (ventral and dorsal) [12,27,28,29,30,31,32,33,34,35,36,37] (Figure 4). In pancreaticobiliary union, the main pancreatic duct joins together with the common bile duct, and they drain into the second portion of the duodenum, but variations can occur, which can be classified into three categories: V type, where the pancreatic duct and common bile duct enter the duodenal wall without a common duct; B–P type, where the common bile duct drains into the pancreatic duct main, forming a common duct; and P–B type, where the main pancreatic duct drains into the bile duct, forming a common duct [11,13,34,37].

Other variations in the pancreatic ducts have been described. Gonoi et al. (2011) described the retroportal main pancreatic duct (RMPD), where the main pancreatic duct runs behind the portal vein; other studies have evaluated the course of the pancreatic duct, and the variants used were descending type, sigmoid type, vertical type, and loop type [16,18]. Moreover, two articles were found that made reference to the vascular variations associated with the pancreas that can occur [24,38]. Yilmaz and Celik (2018) found 55 cases identified with CP, which could be classified according to their relationship with the splenoportal confluence into suprasplenic CP, infrasplenic CP, and mixed CP; they also found other vascular variations in a cadaver which had a duplication of the left gastro-omental (gastroepiploic) artery, in addition to having a vulnerable intrapancreatic tract [38] (Figure 2, Figure 3 and Figure 4).

### 3.2. Characteristics Reported in the Articles

A total of 55 studies were analyzed, which we will describe as follows: type of study, geographical distribution, sex, incidence of variation in the pancreas, and, finally, statistical values reported by the study, seen in Table 1.

For the study type characteristic, 12 were case studies, 9 studies were retrospective, 1 study was a case series, 2 studies were conference abstracts, 1 study was prospective, and 3 studies were observational, showing a wide variety of methodological designs included in this review. Regarding the geographical distribution of the studies, 10 studies were carried out in Europe, 7 in America, 11 in Asia, and, finally, there were no studies in Oceania or Africa. Regarding the sex of the patients, of the 55 studies, 33 did not specify the sex of the patients, and 12 had only men or only women, of which 6 were only women and 6 were only men. It should be noted that these 12 studies were case reports. Of the total studies, 9 included men and women; for men, the incidence varied between 20.6% and 58%, with a mean of 46.5%, while, for women, it varied between 43.3% and 79.4%, with an average of 53.5%.

Regarding the incidence of anatomical variants in the studies, 14 of these presented a 100% variation, since they were case studies, while 2 studies also had an incidence of 100% even when their sample was greater than one: the first of them was a retrospective study with 68 patients with 100% PD; and the second was an observational study with 63 patients with 100% pancreatobiliary junction. Of the 14 studies with a sample of only one individual (N = 1), 2 studies reported CP variation, 3 studies reported bifid pancreas, 1 study reported pancreatic loop, 1 study reported the duplication of the left gastro-omental (gastroepiploic) artery, 4 studies reported PD, 1 study reported annular portal pancreas, 1 study presented the presence of a closed loop of the main pancreatic duct, and 1 study presented variations in the pancreaticobiliary junction. Meanwhile, for the articles with a larger sample (N > 1), the incidence was as follows: circumportal pancreas was reported in 3 studies with the following incidences: 0.8% (6.813), 1.8% (508), and 0.009% (22,628), which have an average of 0.87% incidence; variation of the pancreatic duct was reported in 3 studies with the following incidences: 55% (1158), 56.4% (582), and 78.9% (19), with an average of 63.43%; pancreas divisum was reported in 6 studies with the following incidences: 25% (8), 2.7% (1.529), 1% (100), 50% (274), 5.7% (5.357), and 4.7% (1.158), with an average of 14.85%; annular pancreas was reported in 1 study with an incidence of 8% (50); variations in the contour and head of the pancreas were presented in 1 study, with an incidence of 35% (119); and the length of the pancreatic duct was reported in 1 study with an incidence of 46.8% (310).

Finally, 7 studies [16,23,27,28,32,40,41] showed statistical values in their results, which we will detail below: In the study by Sherifi et al. (2018) [41], no statistical significance was found in relation to the size of the pancreatobiliary angle (PB) according to gender (*p* = 0.633) or age (*p* = 0.792). Ohtsuka et al. (2016) [23] found no significant differences between patients with or without CP in relation to age, sex, pancreatic loop, hepatic artery variations, intraoperative factors, or postoperative complications (*p* = 0.603). Patients with CP had a higher frequency of bile duct cancer (*p* = 0.03); in addition, they had a higher frequency of pancreatic fistula compared to a normal pancreas (*p* = 0.03). Morgan et al. (2008) [32] reported that there were no statistically significant differences in patients who presented a good or poor response to surgery and had PD (*p* = 0.5). Kubota et al. (1993) [40] observed that separate drains from both the main pancreatic and common bile ducts reached the duodenum separately, which was frequently associated with patients with choledocholithiasis (*p* < 0.01). In the study by Delhaye et al. (1985) [28], there was a statistically significant clinical correlation between PD with chronic pancreatitis (*p* < 0.001) and acute pancreatitis (*p* < 0.05). In the study by Bang et al. (2006) [27], the rates of hyperamylasemia in pancreatic ductal Types C and D were significantly higher than in Types A and B (*p* = 0.018) according to the classification proposed by Cubilla et al. (1984) [63]. Finally, Adibelli et al. (2016) indicated that the female–male ratio was 1.36 in favor of women. The female–male ratios of pancreas with a Type II configuration in relation to the variation in the ductal configuration of the pancreas proposed in the study showed a *p*-value of 0.03. The female–male ratio of the vertical course was 0.0048. The gender distributions among the other configuration types did not show statistically significant values: Type 1, *p* = 0.35; Type 2, not described; Type 3, *p* = 0.80; Type 4, *p* = 0.29; and Type 5, *p* = 0.40 [16,43,62].

### 3.3. Prevalence and Risk of Bias

**Figure 5:**
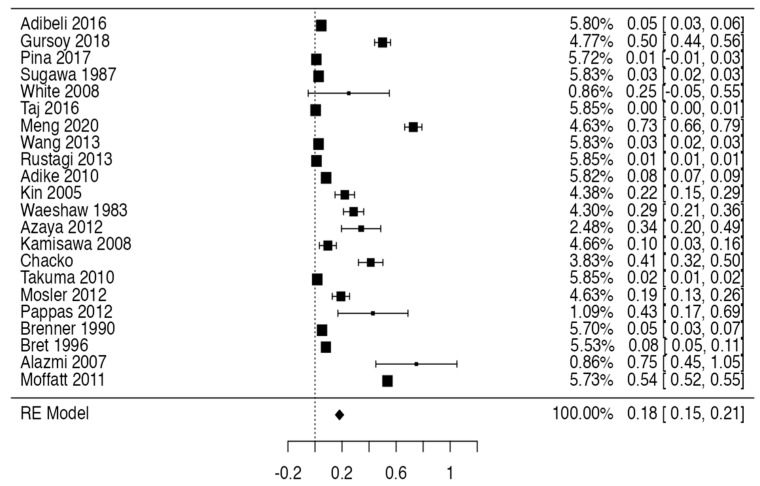
Forest plot of prevalence of PD [7,12,14,16,29,34,36,40,45,46,47,48,49,50,51,52,53,54,57,61,64,65].

**Figure 6:**
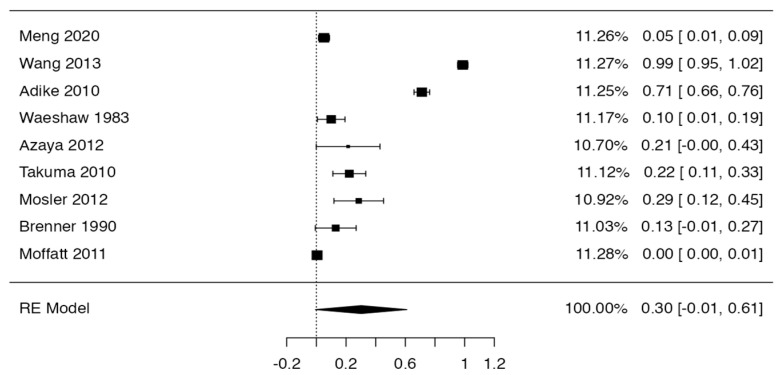
Forest plot prevalence of pancreatitis in PD [43,44,46,54,55,57,62,64].

**Figure 7:**
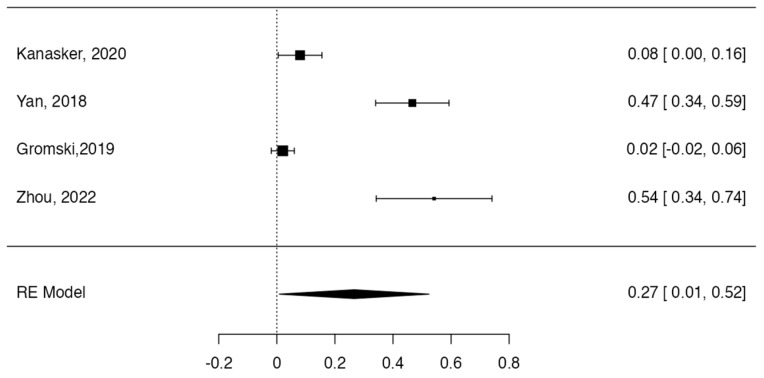
Forest plot prevalence of pancreas annular [11,15,24,66].

**Figure 8:**
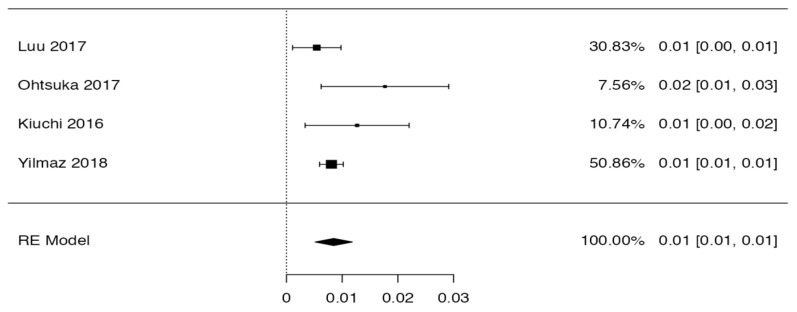
Forest plot of circumportal pancreas [23,24,25,26].

**Figure 9:**
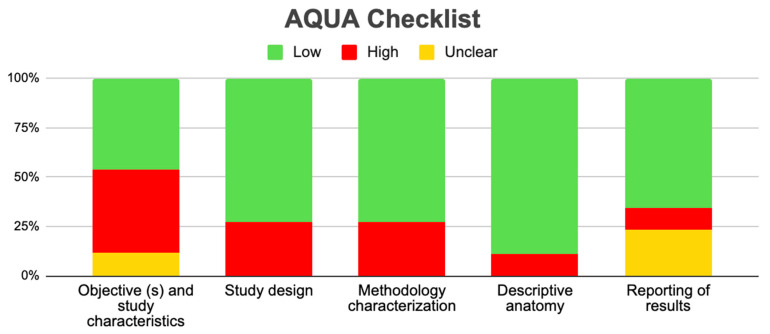
AQUA graphic.

Tree forest plots were carried out to see the prevalence of the variants of the ductal system of the pancreas, and a forest plot was also carried out to see the clinical correlation with PD. For the PD variant, 22 studies [7,12,14,16,29,34,36,40,45,46,47,48,49,50,51,52,53,54,57,61,64,65] presented a prevalence of 0.18 (0.15–0.21) and a heterogeneity of 99.52%. For the annular pancreas prevalence forest plot, 4 studies [11,15,24,66] were included, presenting a prevalence of 0.27 (0.01–0.5) and 97.18% heterogeneity. For the circumportal pancreas forest plot, 4 studies were included [23,24,25,26], presenting a prevalence of 0.01 (0.01–0.01) and a heterogeneity of 97.18%. Finally, a forest plot was performed for the prevalence of pancreatitis on PD, which included 9 studies [43,44,46,54,55,57,62,64] presenting a prevalence of 0.31 (0.01–0.61) and a heterogeneity of 99.78% (Figure 5, Figure 6, Figure 7 and Figure 8). For the risk of bias, five domains were included according to the AQUA classification: for the domain of objectives and characteristics of the included studies, the highest percentage of studies presented a high risk of bias; for the study design domain, the largest number of studies presented a low risk of bias; for the methodological characteristics domain, most of the studies presented a low risk of bias; for the anatomical description domain, most of the studies also presented a low risk of bias; and, finally, the reporting of results domain also mostly presented a low risk of bias (Table 2 and Figure 9).

### 3.4. Clinical Considerations

The anatomical variations of the pancreas can present varied clinical associations, which may depend directly on the anatomical variation and, also, on factors extrinsic to them. Next, we will describe different pathological conditions of the pancreas and why anatomical variations could be exacerbated or conditioned. Acute pancreatitis is an intracellular calcium disorder in pancreatic cells, which can trigger necro-inflammatory changes and local and systemic complications [67]. Within our study, we found different anatomical variations that could produce pancreatitis; PD was one of them, which was reported by eight articles [16,28,31,32,34,35,37,53]. These studies have as a common denominator where the presence of PD could be a predisposing factor for pancreatitis, but none of them reported that this relationship is absolute. Ansa pancreatica could also be considered one of the predisposing factors for pancreatitis, which was described in two articles included in our search [16,26]. Variations in the pancreatic ducts were also reported as determining factors for the development of some types of pancreatitis [27]. The annular-type pancreatic variation also demonstrated the possibility of producing pancreatitis, duodenal obstruction, and other conditions [68]. Bifid pancreas also demonstrated in two studies the possibility of producing obstructive pancreatitis [19,20].

Another pathological condition is the formation of bile duct stones; these are divided according to their location into two types: primary and secondary. They are considered primary when they remain where they formed and secondary when they form in the gallbladder and migrate to the bile duct. The primary ones are subdivided into intrahepatic and extrahepatic, and the limit is the union of the right and left hepatic ducts [11]. Variations in the biliary pancreatic ductal system or the common bile duct were associated with their etiological importance in the formation of gallstones [40].

The anatomical variations of the pancreas can produce preoperative, intraoperative, and postoperative clinical complications in patients who present them. Within our research, 10 studies exposed the importance of recognizing these pancreatic variations to avoid complications such as erroneous diagnoses or a misinterpretation of the pancreas variant; also, intraoperative vascular damage and postoperative pancreatic fistula could occur [2,12,19,21,22,23,24,28,39,42,56,57,58,59,60,61,62,69].

## 4. Discussion

This review aimed to know the anatomical variants of the ductal system, their prevalence, and their association with clinical conditions of the pancreas. Therefore, we analyzed different studies with the aforementioned criteria. The extracted data were grouped according to variations in the ductal system, variations in the pancreaticobiliary junction, and variations in terms of its vascularization, to then be evaluated and statistically analyzed, and, finally, to look for their main clinical correlations. The main results found correspond to a higher prevalence of pancreatitis associated with variants such as PD; in addition, the prevalence in the different variants was high, and, in many of them, there was always a clinical correlation.

Finally, within our search, we found three reviews that met our inclusion criteria. It should be noted that they were studies with a mainly clinical objective, since the clinic was detailed in depth, but a very poor approach was made to the anatomical variant. The differences between our review and the reviews found will be detailed below [56,65,69]. The review carried out by Watson and Harper (2015) was presented as an objective to show the anatomical variations of the pancreas and their association with pancreas transplantation; the study looked for this association not only in the pancreas, but also in other structures such as the kidney, liver, liver ducts, and arteries and veins of the abdominal region, among others. The studies analyzed in this review did not show a relationship between anatomical variations of the pancreas and its transplantation, but we do believe that it is important to know how they might influence this type of surgery. Dimitriou et al. (2018) aimed to show anatomical variations of the ductal system of the pancreas associated with their surgical importance for different procedures performed on this organ. In relation to this, the studies included in our review reported an association between ductal variations and surgical complications such as postoperative pancreatic fistula, but our main objective was to determine how the variants influenced different clinical conditions. The review by Kim et al. (2019), similar to our findings, showed congenital anatomical variations of the pancreas mainly associated with pancreatitis, with the difference that we found a greater number of anatomical variations of the pancreas associated with more clinical conditions that compromise it clinically.

The studies included in this review were mostly case studies and retrospective studies. If we analyze the evidence provided by a case study, it could influence the reproducibility of the data provided by this review, while the proportion of retrospective studies will depend on the risk of bias that these studies present. In relation to the geographical distribution where these studies were carried out, which will be directly related to the sample, it was found that they were carried out mainly in Europe, Asia, and America, which shows regional heterogeneity, which could also be associated with racial heterogeneity in the samples studied. Another important characteristic of the studies is the sex of the sample; this will not be a parameter that we can clearly represent, since, in several studies, the sample was not identified according to sex, which is attributed to the fact that many were carried out on cadaveric samples or cadaveric segments where only the structure or region of interest was analyzed. In relation to this, only nine studies included samples of both sexes, in which no significant difference was found in the percentage of female and male participants, which allows us to infer that this type of variation would not be associated with sex.

Regarding the methodological quality of the studies, this was reviewed with the AQUA checklist for anatomical reviews, where it was found that most of the studies had a high risk of bias, which allows us to say that our results can be extrapolated, applied, and used for new studies, or for informed decision making in the anatomical–clinical field.

In this review, we group the anatomical variations of the pancreas according to variations in the ductal system, variations in the pancreatobiliary junction, and variations in terms of its vascularization. The variations of the pancreatic ductal system are wide, comprising both the main pancreatic duct and the accessory pancreatic duct. One of the main variants is PD, where the main pancreatic duct originates at the level of the neck of the pancreas when it is complete PD, while, in incomplete PD, the main pancreatic duct originates at the level of the neck of the pancreas, but it has a contribution or collateral branch of the accessory pancreatic duct that comes from the tail of the pancreas. The literature describes this anatomical variant as quite frequent, reporting an incidence of 5–10% [70,71]. Annular pancreas is described as an anatomical variant from a rather complex descriptive point of view, since the descending portion of the duodenum is located within the head of the pancreas; on the other hand, the ductal system associated with the head and the neck of the pancreas takes a rather tortuous path around the descending portion of the duodenum to enter the greater and lesser duodenal papillae. This anatomic variant has an incidence of 0.0012% but has been found to increase exponentially to 1.5% in people with Down syndrome [22,31]. The last ductal variation studied corresponded to the loop of the accessory pancreatic duct, in which the accessory pancreatic duct has a lower course, then a higher one, and, finally, goes towards the minor duodenal papilla. Finally, the last description of the variants found is related to the vascular variants. Only two studies [23,24] reported vascular variants associated with the pancreas; these variants are associated with the position of both the splenic artery and the hepatic portal vein, the most common being the splenic artery, which runs inferior to the pancreas, and the portal vein running anterior to the pancreas. Therefore, like PD, these variants must be considered in surgical interventions or diagnostic processes in the abdominal region.

For the statistical data found in this review, the prevalence of PD was assessed in 22 of the total number of studies included, presenting a high prevalence (18%), which is a sign that PD could often be asymptomatic. Meanwhile, four studies included the prevalence of the annular pancreas variant, which was 27%; this indicates that this percentage is overestimated, since the literature reports that this variant is very infrequent and is symptomatic in most of its cases, and so is diagnosed more than the previous ones. The prevalence of the circumportal pancreas variant was 1%, which is consistent with the literature; like PD, this variant is very uncommon and also presents asymptomatically.

On the other hand, the prevalence of pancreatitis in PD based on nine studies was 31%, which, as in previous cases, is high. This could be due to the differences in the definition and diagnosis of pancreatitis, which is prevalent and can be a diagnostic predictor as well as a follow-up pattern for patients with this variant. The clinical, surgical, and diagnostic approach to the pancreas has always been very complex due to the organ’s location. It should be noted that, in many cases, PD will not have clinical implications for the person presenting this variation, but we have shown that the presence of pancreatitis associated with PD is very probable; therefore, knowing the exact mechanisms in this relationship would be very useful for the management and diagnosis of pancreatitis, which may help to make more informed clinical decisions. In other cases, PD may go unnoticed throughout life, but it should be considered in the diagnosis, treatment, or surgical approaches to the abdomen.

The clinical presentation of annular pancreas will depend on the type of annular pancreas, according to Shippen’s classification: (i) there may be complete duodenal obstruction at the level of the head of the pancreas, which requires surgery at birth; (ii) there may be late-onset duodenal obstruction and slowly progressive symptoms during childhood or adolescence, for which surgical intervention will also be necessary at some point; or (iii) minimal or incoherent duodenal obstruction may be present, in which the person will be asymptomatic for life, and this finding could be found mainly at autopsy or cadaveric dissection [72,73]. From a clinical point of view, this variant of the ductal system is the one that will have the fewest implications, and, in most people who present it, it will not have repercussions throughout life [69].

## 5. Limitations

The limitations of this systematic review are the publication bias of the included studies, since studies with different results that were in non-indexed literature in the selected databases may have been overlooked; the probability of not having conducted a most specific and sensitive search in relation to the topic to be studied; and, finally, personal gatherings of the authors for the selection of articles.

## 6. Conclusions

This study shows that the variants in the morphology of the pancreas can be multiple and that they are found, for the most part, in the ductal system. Although the ductal system is the main structure with variants, another variation of great anatomoclinical relevance is annular pancreas, in which the duodenum crosses the head of the pancreas. Although these variants do not have a high population incidence, in this review, we have been able to demonstrate that, if any of the aforementioned variants is present, the probability of presenting a clinical condition increases exponentially in relation to patients who do not present it, with the most classic being pancreatitis. We believe that the professional who deals with the abdominal region should take these variants into account for various diagnostic and treatment approaches.

## Figures and Tables

**Table 1:** Characteristics studies include the following information.

Author(s), Year	Type of Study and Number of Participants (N)	Incidence	Statistical Values	Geographic Region	Sex/Gender
Addeo et al., 2019 [21]	Case study, 1 patient	100% circumportal pancreas	Not presented	France	Female
Adibelli et al., 2016 [16]	Retrospective study, 1158 patients	The anatomical variation in the pancreatic duct was 55%; variations in the course of the pancreatic duct and ansa pancreatica was 37.5%; pancreas divisum of 4.7%; and 2.8% was without variants.	Female–male ratio was 1.36. The proportions of type II configuration showed a value of 0.03 (IS), while the ratio of the vertical course was 0.0048 (IS).	Turkey	490 males (42.3%) and 668 females (57.7%)
Bang et al., 2006 [27]	Retrospective study, 582 patients	The anatomical variation of the pancreatic ductal system was 56.4%.	The rates of hyperamylasemia in types Cyd were significantly higher than in types A and B (*p* = 0.018).	Republic of Korea	325 males (55.8%) and 257 females (44.2%)
Delhaye et al., 1985 [28]	Case series, 5357 patients	The pancreas divisum was present in 5.7% of patients.	Significant correlation between PD with chronic pancreatitis (*p* < 0.001) and acute pancreatitis (*p* < 0.05).	Belgium	Not specified
Qin et al., 2019 [12]	Case study, 1 patient	100% incidence for portal annular pancreas.	Not presented	China	Female
Yang et al., 2019 [13]	Retrospective study, 60 patients with ERCP	28 patients with AP.	2 of 28 patients presented duodenal obstruction.	China	Not specified
Zhou et al., 2022 [14]	Retrospective study, 24 patients with ERCP	13 patients with AP.	2 patients with pancreatitis.	China	10 males and 14 females
Gromski et al., 2019 [15]	Prospective study, 49 patients with ERCP	1 patients with AP.	Does not present	Poland	Not specified
Halpert et al., 1990 [20]	Case study, 1 patient	100% incidence of bifid pancreas.	Not presented	USA	Female
Ishida et al., 2019 [2]	Case study, 1 patient	100% incidence of bifid pancreas.	Not presented	Japan	Female
Jarrar et al., 2013 [17]	Case study, 1 patient	100% incidence of pancreatic ansa.	Not presented	France	Male
Kanasker and Bharambe, 2016 [11]	Congress summary, 50 corpses	Incidence of 4% complete annular pancreas and 4% incomplete annular pancreas.	Not presented	India	Not mentioned
Gonoi et al., 2011 [22]	Retrospective study, 22,628 patients	The incidence of circumportal pancreas was 2 patients (0.009).	Not reported	Japan	Not reported
Ohtsuka et al., 2016 [23]	Retrospective study, 508 patients	The incidence was 9 patients with circumportal pancreas, which is equivalent to 1.8%.	There were no significant differences in age, sex, and ASA. Patients with circumportal pancreas have a more frequent diagnosis of bile duct cancer (*p* = 0.03) and a higher frequency of pancreatic fistula compared with a normal pancreas (*p* = 0.03).	Japan	293 males (57.7%) and 215 females (42.3%)
Yilmaz and Celik, 2018 [24]	Retrospective study	0.8% incidence for circumportal pancreas.	Does not present statistical values	Turkey	Does not fully report the sex of those studied
Luu et al., 2017 [25]	Retrospective study, 1102 patients through surgical analysis	6 patients with CP.	Does not present	Germany	368 males and 734 females
Kumar et al., 2019 [38]	Case study, 1 cadaver	100% incidence of duplication of the left gastroepiploic (gastro-omental) artery.	Not presented	France	Male
Delhaye et al., 1985 [28]	Retrospective study, 5347 cholangiopancreatography retrograde	304 patients with PD.	Does not present	Belgium	147 males and 157 females
Montagnani et al., 2013 [30]	Case study, 1 patient	100% incidence for PD.	Not presented	Italy	Male
Morgan et al., 2008 [32]	Retrospective study, 68 patients	100% incidence for PD.	There was no significance in the patients with PD and their response to surgery (*p* = 0.5).	USA	14 males (20.6%) and 54 females (79.4%)
Nahmod et al., 2017 [33]	Case study, 1 patient	100% incidence for PD.	Not presented	Argentina	Male
Pina et al., 2017 [34]	Case series study, 100 cadavers	Incidence of 1 pancreas divisum with 1%.	Not presented	Argentina	Not specified
Sanada et al., 1995 [35]	Case study	100% incidence for pancreas divisum.	Not presented	Japan	Male
Sugawa et al., 1979 [36]	Retrospective study, 1529 patients	2.7% incidence for PD.	Not presented	USA	Not registered
White et al., 2014 [37]	Observational study; 8 patients	The incidence for pancreas divisum was 25%.	Does not present statistical values	USA	4 males (50%) and 4 females (50%)
Ross et al., 1996 [39]	Retrospective study, 119 patients	35% incidence regarding variations in the contour of the head and neck of the pancreas.	Not presented	USA	69 males (58%) and 50 females (42%)
Kubota et al., 1993 [40]	Prospective study, 310 patients	Percentages for common duct length: 7.7 ± 2.1 mm 48 (15.5%); 7.8 ± 3.1 mm 20 (6.5%); 8 ± 2.7 mm 77 (24.8%).	Patients with choledocholithiasfrequently present separate drainage of both ducts in the duodenum (*p* < 0.01).	Japan	138 males (44.5%) and 172 females (55.5%)
Sherifi et al., 2018 [41]	Observational study; 63 patients studied	The incidences were: 31.7% of the BP type, 30.2% had a pathology that deforms the PB junction, 28.6% of the duodenal type, 7.9% of the PB type, and 1.6% presented artifacts.	Without statistical significance into the size of P–B according to sex (*p* = 0.633). No correlation was found between age and the size of the P–B angle (*p* = 0.792).	Kosovo	32 males (50.8%) and 31 females (49.2%)
Singh et al., 2017 [42]	1 case study	100% incidence in the presence of a closed loop of the main pancreatic duct.	Not reported	India	Male
Moffatt et al., 2011 [43]	Retrospective study, 2753 patients with ERCP	1476 patients with PD.	Does not present	Germany	Not specified
Adike et al., 2010 [44]	Retrospective study, 3456 patients	284 patients with pancreas divisum.	82 without pancreatitis and 202 with pancreatitis *p* = 0.008.	USA	108 males and 176 females
Alazmi et al., 2007 [45]	Case series, 80 patients evaluated with ERCP	6 patients with PD.	Does not present	USA	Not present
Tajima et al., 2009 [19]	Case study	100% incidence in relation to bifid pancreatic duct.	Not presented	Japan	Female
Brenner et al., 1990 [46]	Retrospective study, 441 pancreatography	23 patients with PD.	Does not present	France	Not specified
Chacko et al. 2008 [47]	Retrospective study, 114 patients	47 patients with PD.	Does not present	USA	Not specified
Kamisawa et al., 2008 [48]	Case series, 84 patients	8 patients with PD.	1 patient with PD was associated with gallbladder cancer.	China	Not specified
Kin et al., 2005 [49]	Case series, 127 patients	28 patients with PD.	Does not present	Canada	67 males and 60 females
Meng et al., 2020 [50]	Retrospective study, 187 patients	136 patients with pancreas divisum.	15.7% without pancreas divisum presented pancreatitis vs. 5.6% with pancreas divisum presented pancreatitis, *p* = 0.005)	China	107 males (57%) and 80 females (43%)
Pappas et al., 2012 [51]	Retrospective study, 14 patients	6 patients with PD.	Does not present	USA	5 males and 9 females
Rustagi et al., 2013 [52]	Prospective study, 4121 patients	45 patients with pancreas divisum.	Does not present	USA	23 males and 25 females
Sugawa et al., 1987 [53]	Retrospective study, 1529 pancreatography	41 pancreatography presented PD.	17 of 41 PD presented pancreatitis.	USA	Not specified
Takuma et al., 2010 [54]	Case series, 3246 patients	54 patients with complete PD; 50 patients with incomplete PD.	12 of 54 patients with PD presented pancreatitis *p* = 0.01.	Japan	Not specified
Wang et al., 2013 [55]	Retrospective study, 1439 patients	38 patients with PD.	Does not present	China	698 males (49%) and 741 females (51%)
Kim et al., 2018 [56]	Case study, 1 patient	The reported incidence was 100% circumportal pancreas accompanied by PD.	Not presented	France	Female
Mosler et al., 2012 [57]	Case series, 146 patients	28 patients with PD.	8 of 28 patients with PD presented pancreatitis.	USA	Not specified
Bret et al., 1996 [58]	Retrospective study, 310 pancreatography	25 patients with PD.	Does not present	Canada	Not specified
Malathi et al., 2017 [59]	Congress summary, 19 corpses	The incidence was 13 adult patients with ansa pancreatica (68.4%), and 2 embryonic type patients (10.5%).	Not presented	India	Not specified
Tappouni et al., 2015 [60]	44 case studies through CT scan	37 patients with CP.	2 patients with pancreatitis.	USA	13 males and 31 females
Taj et al., 2016 [61]	Retrospective study, 3600 patients	17 pancreas divisum (0.47%).	Does not present statistical values	Saudi Arabia	report the sex of those studied
Warshaw et al., 1983 [62]	Case series, 140 patients	40 patients with PD.	4 patients with PD presented pancreatitis *p* = 0.01.	USA	13 males and 27 females

**Table 2:** AQUA tool application and assessment details.

References	Study	Domain 1				Domain 2					Domain 3					Domain 4						Domain 5				
		1	2	3	4	5	6	7	8	9	10	11	12	13	14	15	16	17	18	19	20	21	22	23	24	25
Addeo et al., 2019 [21]	Retrospective study	Y	Y	N	Y	Y	Y	Y	Y	Y	Y	Y	Y	Y	Y	Y	Y	Y	Y	Y	Y	Y	Y	Y	Y	Y
Adibelli et al., 2016 [16]	Retrospective study	U	Y	U	N	Y	Y	U	Y	Y	U	Y	N	N	N	N	N	Y	N	N	N	Y	Y	Y	NA	Y
Bang et al., 2006 [27]	Cadaveric study	N	Y	U	N	Y	Y	N	Y	Y	Y	N	N	N	Y	Y	N	N	Y	N	N	Y	Y	Y	NA	Y
Delhaye et al., 1985 [28]	Cadaveric study	Y	N	N	N	Y	Y	Y	N	Y	N	Y	Y	Y	Y	Y	N	N	N	Y	N	Y	Y	N	NA	Y
Qin et al., 2019 [12]	Cadaveric study	Y	Y	N	N	Y	N	Y	Y	Y	N	Y	N	N	Y	Y	Y	Y	Y	N	N	Y	Y	Y	NA	Y
Yang et al., 2019 [13]	Cadaveric study	Y	N	N	Y	N	Y	Y	Y	Y	Y	N	N	Y	N	Y	N	Y	Y	N	Y	Y	Y	N	NA	Y
Zhou et al., 2022 [14]	Retrospective study	Y	Y	Y	N	Y	Y	Y	Y	N	Y	Y	N	Y	N	Y	Y	Y	Y	Y	N	Y	Y	Y	NA	Y
Gromski et al., 2019 [15]	Retrospective study	Y	Y	Y	Y	N	Y	Y	Y	Y	Y	Y	Y	N	Y	Y	Y	Y	Y	N	Y	Y	Y	Y	NA	Y
Halpert et al., 1990 [20]	Cadaveric study	Y	N	Y	N	Y	N	N	Y	N	Y	Y	N	Y	N	Y	N	Y	N	N	Y	Y	Y	Y	NA	Y
Ishida et al., 2019 [2]	Cadaveric study	U	Y	N	Y	N	Y	Y	N	N	Y	Y	N	N	N	Y	N	N	Y	N	Y	Y	Y	Y	NA	N
Jarrar et al., 2013 [17]	Cadaveric study	U	Y	N	N	Y	Y	N	Y	N	Y	N	Y	Y	N	Y	Y	Y	Y	N	Y	Y	Y	Y	NA	Y
Kanasker and Bharambe, 2016 [11]	Cadaveric study	Y	N	Y	N	Y	N	Y	Y	Y	N	Y	Y	N	Y	Y	N	N	Y	N	Y	N	Y	Y	NA	Y
Gonoi et al., 2011 [22]	Cadaveric study	Y	N	N	Y	N	Y	N	Y	Y	N	Y	N	Y	N	Y	Y	N	N	Y	N	Y	Y	Y	NA	Y
Ohtsuka et al., 2016 [23]	Cadaveric study	N	Y	Y	Y	N	Y	N	Y	N	N	Y	Y	N	N	Y	N	N	Y	N	Y	Y	Y	Y	NA	Y
Yilmaz and Celik, 2018 [24]	Cadaveric study	Y	Y	Y	Y	N	Y	N	N	N	N	Y	Y	N	N	Y	N	N	Y	Y	Y	Y	Y	Y	NA	Y
Luu et al., 2017 [25]	Cadaveric study	Y	N	N	Y	Y	Y	Y	Y	N	N	Y	N	N	N	Y	N	N	Y	N	Y	Y	Y	Y	NA	Y
Kumar et al., 2019 [38]	Retrospective study	Y	Y	Y	Y	Y	N	Y	Y	Y	Y	Y	N	Y	N	Y	N	Y	Y	Y	Y	Y	Y	Y	NA	N
Delhaye et al., 1985 [28]	Retrospective study	Y	Y	Y	Y	Y	N	Y	Y	Y	Y	Y	Y	Y	N	Y	N	Y	N	Y	Y	Y	Y	Y	NA	N
Montagnani et al., 2013 [30]	Retrospective study	Y	Y	Y	Y	Y	N	Y	Y	Y	Y	Y	Y	Y	N	Y	N	Y	N	Y	Y	Y	Y	Y	NA	N
Morgan et al., 2008 [31]	Retrospective study	Y	Y	Y	Y	Y	N	Y	Y	Y	Y	Y	N	Y	N	Y	N	Y	Y	Y	Y	Y	Y	Y	NA	N
Nahmod et al., 2017 [33]	Cadaveric study	Y	Y	Y	N	Y	N	Y	N	Y	N	N	Y	Y	Y	N	Y	N	Y	Y	N	Y	Y	Y	NA	Y
Pina et al., 2017 [34]	Retrospective study	Y	N	Y	Y	Y	N	Y	Y	Y	Y	Y	N	Y	N	Y	N	Y	Y	Y	Y	Y	Y	Y	NA	Y
Sanada et al., 1995 [35]	Retrospective study	Y	Y	Y	Y	Y	N	Y	Y	Y	Y	Y	Y	Y	N	Y	N	Y	N	Y	Y	Y	Y	Y	NA	N
Sugawa et al., 1979 [36]	Cadaveric study	U	Y	N	N	Y	Y	N	Y	N	Y	N	Y	Y	N	Y	Y	Y	Y	N	Y	Y	Y	Y	NA	Y
White et al., 2014 [37]	Cadaveric study	Y	N	Y	N	Y	N	Y	Y	Y	N	Y	Y	N	Y	Y	N	N	Y	N	Y	N	Y	Y	NA	Y
Ross et al., 1996 [39]	Cadaveric study	Y	Y	Y	N	Y	N	Y	N	Y	N	N	Y	Y	Y	N	Y	N	Y	Y	N	Y	Y	Y	NA	Y
Kubota et al., 1993 [40]	Cadaveric study	Y	N	N	Y	N	Y	N	Y	Y	N	Y	N	Y	N	Y	Y	N	N	Y	N	Y	Y	Y	NA	Y
Sherifi et al., 2018 [41]	Retrospective study	Y	Y	Y	N	Y	Y	Y	N	Y	Y	Y	Y	Y	N	Y	Y	N	Y	N	Y	Y	Y	Y	NA	B
Singh et al., 2017 [42]	Retrospective study	Y	Y	Y	Y	Y	Y	Y	N	Y	Y	Y	N	Y	Y	N	Y	Y	Y	Y	N	N	Y	Y	NA	Y
Moffatt et al., 2011 [43]	Prospective study	Y	N	Y	Y	Y	Y	Y	Y	N	Y	Y	Y	Y	N	Y	Y	Y	Y	Y	N	Y	Y	Y	NA	N
Adike et al., 2010 [44]	Retrospective study	Y	Y	Y	N	Y	N	N	Y	Y	Y	Y	N	Y	N	Y	Y	Y	Y	Y	Y	N	Y	Y	NA	Y
Alazmi et al., 2007 [45]	Case series	Y	N	Y	Y	N	N	Y	Y	N	Y	Y	Y	Y	Y	N	Y	Y	Y	N	Y	Y	N	N	NA	Y
	Case series	Y	Y	Y	Y	Y	Y	Y	N	N	Y	N	Y	Y	Y	N	Y	N	N	Y	Y	Y	Y	N	NA	Y
Tajima et al., 2009 [19]	Prospective study	Y	N	Y	Y	N	N	Y	Y	N	Y	Y	Y	Y	Y	N	Y	Y	Y	N	Y	Y	N	N	NA	Y
Brenner et al., 1990 [46]	Case series	Y	Y	Y	Y	Y	Y	Y	N	N	Y	N	Y	Y	Y	N	Y	N	N	Y	Y	Y	Y	N	NA	Y
Chacko et al. 2008 [47]	Retrospective study	Y	N	Y	Y	Y	N	Y	Y	Y	Y	Y	N	Y	N	Y	N	Y	Y	Y	Y	Y	Y	Y	NA	Y
Kamisawa et al., 2008 [48]	Case series	Y	Y	Y	N	Y	Y	Y	Y	N	N	Y	N	Y	N	Y	Y	Y	N	Y	Y	Y	Y	Y	NA	N
Kin et al., 2005 [49]	Case series	Y	Y	Y	Y	N	Y	Y	Y	N	N	Y	Y	Y	N	Y	Y	Y	Y	N	Y	Y	Y	Y	NA	N
Meng et al., 2020 [50]	Retrospective study	Y	Y	Y	N	Y	Y	Y	N	Y	Y	Y	Y	Y	N	Y	Y	N	Y	N	Y	Y	Y	Y	NA	B
Pappas et al., 2012 [51]	Retrospective study	Y	Y	Y	Y	Y	Y	Y	N	Y	Y	Y	N	Y	Y	N	Y	Y	Y	Y	N	N	Y	Y	NA	Y
Rustagi et al., 2013 [52]	Retrospective study	Y	N	Y	Y	Y	Y	Y	Y	N	Y	Y	Y	Y	N	Y	Y	Y	Y	Y	N	Y	Y	Y	NA	N
Sugawa et al., 1987 [53]	Retrospective study	Y	Y	Y	N	Y	N	N	Y	Y	Y	Y	N	Y	N	Y	Y	Y	Y	Y	Y	N	Y	Y	NA	Y
Takuma et al., 2010 [54]	Retrospective study	Y	Y	Y	Y	Y	N	Y	Y	Y	Y	Y	Y	Y	N	Y	N	Y	N	Y	Y	Y	Y	Y	NA	N
Wang et al., 2013 [55]	Case series	Y	Y	Y	Y	Y	N	Y	Y	Y	Y	Y	N	Y	N	Y	N	Y	Y	Y	Y	Y	Y	Y	NA	N
Kim et al., 2018 [56]	Retrospective study	Y	Y	Y	Y	Y	Y	Y	N	Y	Y	Y	N	Y	Y	N	Y	Y	Y	Y	N	N	Y	Y	NA	Y
Mosler et al., 2012 [57]	Retrospective study	Y	Y	Y	N	Y	Y	Y	N	Y	Y	Y	Y	Y	N	Y	Y	N	Y	N	Y	Y	Y	Y	NA	B
Bret et al., 1996 [58]	Prospective study	Y	Y	Y	Y	Y	Y	Y	N	Y	Y	Y	N	Y	Y	N	Y	Y	Y	Y	N	N	Y	Y	NA	Y
Malathi et al., 2017 [59]	Retrospective study	Y	N	Y	Y	Y	Y	Y	Y	N	Y	Y	Y	Y	N	Y	Y	Y	Y	Y	N	Y	Y	Y	NA	N
Tappouni et al., 2015 [60]	Retrospective study	Y	Y	Y	N	Y	N	N	Y	Y	Y	Y	N	Y	N	Y	Y	Y	Y	Y	Y	N	Y	Y	NA	Y
Taj et al., 2016 [61]	Retrospective study	Y	Y	Y	Y	Y	Y	Y	N	Y	Y	Y	N	Y	Y	N	Y	Y	Y	Y	N	N	Y	Y	NA	Y
Warshaw et al., 1983 [62]	Case series	Y	Y	Y	Y	Y	N	Y	Y	Y	N	Y	N	Y	N	Y	N	Y	Y	Y	Y	Y	Y	Y	NA	Y

The characteristics of each domain in the table are available in the article of the author Henry et al. [9].

## Data Availability

Not applicable.

## Supplementary Materials

The following supporting information can be downloaded at: https://www.mdpi.com/article/10.3390/life13081710/s1, Table S1: Search strategies.
