# Supplementary material for: Incidence and Clinical Implications of Anatomical Variations in the Pancreas and Its Ductal System: A Systematic Review and Meta-Analysis
Source: Life (Basel). 2023 Aug 9;13(8):1710. doi: 10.3390/life13081710 (PMC10455790; doi:10.3390/life13081710)
Supplement: Supplementary file 1 [file life-13-01710-s001.zip › life-2525896-supplementary.pdf]

Supplemental Digital Content. Table S1. Searches strategies

Database	Search strategy	Results	
		07-07-23	
**Medline**	Search: **((((ductal pancreas anatomical variations)) OR (Pancreas divisum)) AND (pancreatitis)) NOT (animals)** ((("ductal"[All Fields] AND ("pancrea"[All Fields] OR "pancreas"[MeSH Terms] OR "pancreas"[All Fields]) AND ("anatomic"[All Fields] OR "anatomical"[All Fields] OR "anatomically"[All Fields]) AND ("variation"[All Fields] OR "variations"[All Fields])) OR ("pancreas divisum"[MeSH Terms] OR ("pancreas"[All Fields] AND "divisum"[All Fields]) OR "pancreas divisum"[All Fields])) AND ("pancreas"[MeSH Terms] OR "pancreas"[All Fields] OR "pancreatic"[All Fields] OR "pancreatitides"[All Fields] OR "pancreatitis"[MeSH Terms] OR "pancreatitis"[All Fields])) NOT ("animals"[MeSH Terms:noexp] OR "animals"[All Fields])	985	
**SCOPUS**	Search: ((((ductal pancreas anatomical variations)) OR (Pancreas divisum)) AND (pancreatitis)))	123	
**Google scholar**	Search: ((((ductal pancreas anatomical variations)) OR (Pancreas divisum)) AND (pancreatitis))	231	
**CINHAL**	Search: ((((ductal pancreas anatomical variations)) OR (Pancreas divisum)) AND (pancreatitis))	42	

**WOS**	Search: ((((ductal pancreas anatomical variations)) OR (Pancreas divisum)) AND (pancreatitis))	112	
	Total	1493	

* All searches were carried out on July 07, 2023.
